# Cardiovascular Disease Subtypes and Alzheimer's Disease: Phenotypic and Genetic Associations in the UK Biobank and All of Us Research Program

**DOI:** 10.1161/JAHA.125.046172

**Published:** 2026-06-10

**Authors:** Aili Toyli, Chen Zhao, Kuan‐Jui Su, Hui Shen, Hong‐Wen Deng, Qing‐Hui Chen, Qiuying Sha, Weihua Zhou

**Affiliations:** ^1^ Department of Mathematical Sciences Michigan Technological University Houghton MI USA; ^2^ Department of Computer Science Kennesaw State University Marietta GA USA; ^3^ Division of Biomedical Informatics and Genomics, Tulane Center of Biomedical Informatics and Genomics, Deming Department of Medicine Tulane University New Orleans LA USA; ^4^ Department of Kinesiology and Integrative Physiology Michigan Technological University Houghton MI USA; ^5^ Department of Applied Computing Michigan Technological University Houghton MI USA; ^6^ Center for Biocomputing and Digital Health Institute of Computing and Cybersystems, and Health Research Institute, Michigan Technological University Houghton MI USA

**Keywords:** Alzheimer's disease, cardiovascular disease, cerebral infarction, heart–brain axis, hypotension, Cardiovascular Disease, Risk Factors, Aging

## Abstract

**Background:**

Cardiovascular disease (CVD) and Alzheimer's disease (AD) are major public health concerns that share overlapping risk factors and potential mechanistic pathways. Although vascular contributions to cognitive decline are well documented, the specific relationships between AD and different CVD subtypes remain poorly understood.

**Methods:**

In this cross‐sectional study, we examined associations between AD and 11 CVD subtypes using logistic regression models in 2 large biobanks: the UK Biobank (n=502 133) and the All of Us Research Program (n=287 011). Models were adjusted for demographic, lifestyle, and clinical covariates. We also explored genetic overlap between AD and CVD traits through proximity‐based analysis of significant single nucleotide variants (*P*<5 × 10^−8^) using genome‐wide association study data.

**Results:**

Most CVD subtypes were significantly associated with AD in both cohorts. Hypotension had the strongest and most consistent association, although it has been comparatively understudied in AD research. Strong associations were also consistently observed between AD and hypertension and cerebral infarction. Notably, acute myocardial infarction was not significantly linked to AD. Genetic analyses revealed shared loci between AD‐ and CVD‐related traits, particularly in regions near *APOE*, *MAPT*, and genes influencing myocardial structure and vascular function.

**Conclusions:**

This study identifies subtype‐specific CVD associations with AD across 2 diverse cohorts and highlights shared genetic architecture underlying heart–brain interactions. These findings underscore the importance of vascular health in AD risk and suggest that certain CVD subtypes, especially hypotension, may play underrecognized roles in cognitive decline.

**Nonstandard Abbreviations and Acronyms**
AD: Alzheimer's disease
AoU: All of Us Research Program
GWAS: genome‐wide association study
UKB: UK Biobank

**Research Perspective**

**What Is New?**

- Most cardiovascular disease subtypes were significantly associated with Alzheimer's disease in an analysis of 2 distinct data sets.
- Despite being understudied compared with other subtypes, hypotension consistently exhibited the strongest association with Alzheimer's disease, suggesting the importance of future research into this relationship.

**What Question Should Be Addressed Next?**

- Future studies should use causal inference methods to identify specific pathways that may be targeted to reduce Alzheimer's disease risk.

A growing body of evidence supports a strong and bidirectional relationship between cardiovascular and brain health.^1^ The brain relies heavily on the heart, receiving 15% of the body's cardiac output and 20% of its oxygen supply,^2^ and the heart's function is regulated by the nervous system.^3^ Among the many intersections between these 2 systems, the link between cardiovascular disease (CVD) and Alzheimer's disease (AD)—the most common form of dementia—is of particular interest.^1^, ^2^

Both CVD and AD are age‐associated chronic conditions that share overlapping risk factors including obesity, diabetes, smoking, environment, stress, and sex differences.^4^, ^5^ Pathophysiologically, vascular damage from conditions like hypertension can disrupt the blood–brain barrier and lead to cerebral small vessel disease, manifesting as features such as white matter hyperintensities and microinfarcts,^6^ which are associated with cognitive decline.^7^ Conversely, forms of AD pathology like β‐amyloid plaques and tau deposition may impair regulation of the autonomic nervous system and induce damage to cardiovascular function.^4^, ^8^ Despite this known interplay, the differential impact of specific CVD subtypes on AD risk remains poorly understood.

Large‐scale population biobanks like the UKB (UK Biobank)^9^ and the AoU (All of Us) Research Program^10^ offer an unprecedented opportunity to clarify these relationships through robust, high‐powered analyses across diverse populations.^11^ Prior studies have examined individual CVD traits in relation to cognitive decline or AD, but comprehensive, subtype‐level comparisons are limited.^12^, ^13^, ^14^, ^15^, ^16^ This study aims to fill that gap by examining associations between AD and a wide range of CVD subtypes in both UKB and AoU, while also exploring shared genetic architecture through genome‐wide association study (GWAS) data. Understanding which CVD subtypes are most strongly linked to AD may reveal key mechanistic pathways and inform targeted prevention strategies.

## Methods

### Study Population

In our cross‐sectional study, we used data from 2 large, population‐based biobanks: the UKB and the AoU Research Program. All code has been made publicly available and can be accessed at (https://github.com/MIILab‐MTU/CVDSubtypesADCorrelation). To protect participant privacy, the data are not publicly available; however, they may be accessed by registered users of UKB and AoU. UKB is a prospective cohort of 502 133 participants recruited from 22 centers across the United Kingdom between 2006 and 2010. The UKB resource is open to all bona fide researchers. Full details of its design and conduct are available online (https://www.ukbiobank.ac.uk). UKB received ethical approval from the National Health Service Research Ethics Service (11/NW/0382); we conducted this analysis under application number 61915. All participants provided written informed consent, and the research was conducted in line with the Declaration of Helsinki.

AoU is a US‐based cohort established in 2015, with >746 000 enrolled participants, of whom 287 011 had linked electronic health record and survey data necessary for this study. The AoU Research Program is reviewed by an internal human subjects review board and all participants provide informed consent. Secondary analyses of deidentified data from AoU is not considered human subjects research. Full details of study design and recruitment are available online (https://allofus.nih.gov/article/all‐us‐research‐program‐protocol).

All UKB participants as of December 2024 were included in our analysis (n=502 133). The AoU cohort was limited to participants with linked electronic health record (n=287 011).

### Phenotype Ascertainment

AD and CVD subtypes were identified via *International Classification of Diseases, Tenth Revision* (*ICD‐10*)^17^ codes from electronic health record. Previous studies have found diagnoses obtained from *ICD‐10* codes to have high specificity but lower sensitivity.^18^, ^19^, ^20^ Despite concerns that *ICD‐10* codes may misclassify some diseased patients as healthy, this method was used, as it provides a uniform diagnostic criterion attainable from both data sets.^21^ We included 10 CVD subtypes with ≥10 000 cases in UKB: hypertension (I10), hypotension (I95), angina pectoris (I20), acute myocardial infarction (AMI; I21), pulmonary embolism (I26), cardiac arrhythmia (I44, I48), heart failure (I50), chronic rheumatic heart disease (I05–I09), chronic ischemic heart disease (I25), and cerebral infarction (I63). AD was defined using *ICD‐10* code G30.

### Statistical Analysis

We performed cross‐sectional logistic regression to estimate the odds ratios (ORs) of AD associated with each CVD subtype. Models were adjusted for key covariates affecting heart and brain health: age at assessment, sex, smoking status, education (age completed full‐time education), depression status, physical activity (moderate and vigorous days/week), alcohol consumption, ethnicity, body mass index, annual income, and type 2 diabetes status.^1^, ^4^, ^22^, ^23^

In UKB, most covariates were self‐reported via baseline touchscreen questionnaire at the first visit to the assessment center.^24^ Body mass index was calculated from height and weight measurements at first visit, diabetes status was determined through clinical records (E11), and depression was classified as “Yes” for a positive response to any type of depression or bipolar disorder, and “No” otherwise. All racial subgroups within the “White,” “Black,” “Mixed,” and “Asian” categories were described as their broader classification to ensure sufficient sample size. All other variables retained their original levels from the UK Biobank.^25^ Missing continuous covariate values were imputed using the mean value and categorical missing values were grouped as “Prefer not to answer.”

The same models were applied using AoU data where possible. In AoU, we were not able to calculate the OR for pulmonary embolism, as low AD case counts made estimates highly unstable. Due to limited data availability, physical activity was excluded. Body mass index was averaged from all values reported for each participant. Age was estimated as years since birth as of 2025. Depression and diabetes were identified using *ICD‐10* codes. Racial and ethnic background, smoking, income, alcohol use, and sex at birth were derived from self‐reports, with “Prefer not to answer” and “Skip” responses combined. All individuals who listed multiple ethnicities were classified as “Mixed.”

Sensitivity analyses were performed to ensure statistically robust findings. First, multiple imputation by chained equations was tested in place of mean imputation.^26^ Next, because AD and many CVD subtypes had heavy case–control imbalances, we computed ORs using Firth regression, a method suited for such cases.^27^

We performed adjusted logistic regression with results stratified by race and ethnicity. We condensed several of the strata to ensure sufficient sample size for estimation. In the UKB, “Asian” and “Chinese” were combined under “Asian” and responses of “Mixed,” “Other,” “Unknown,” and “Prefer not to answer” were grouped as “Other.Unknown.” In AoU, “Middle Eastern and North African”, “Native Hawaiian and Pacific Islander,” “Mixed,” “None of these,” and “Prefer not to answer” were grouped as “Other.Unknown.” For AoU, we reported demographic summary statistics across these racial and ethnic classifications to prevent low cell counts and protect participant privacy. We then compared the strength of CVD and AD relationships to determine whether they differ across populations.

Finally, we expanded the analysis to include adjustment for comorbidity of CVD subtypes, because the various forms of CVD frequently co‐occur. Due to concerns of multicollinearity rendering odds ratio estimates unstable, we checked the strength of pairwise correlations between CVD subtype diagnoses.

### Genetic Analysis

To explore shared genetic architecture, we examined proximity between significant single nucleotide variants (SNVs) from UKB CVD GWASs performed by Backman et al.^28^ and SNVs from published GWASs related to brain structure and function included in the National Human Genome Research Institute‐European Bioinformatics Institute catalog^29^ under the topics of dementia and its subtraits, including AD, psychological traits, and traits related to amyloid and tau levels. We assessed the reverse relationship too, as AD GWAS results from UKB were compared with catalog SNVs and cardiovascular disease traits, including traits related to diastolic and systolic blood pressure, ECG derived traits, heart function, cardiac magnetic resonance imaging values, heart rate, and heart shape measurements.

We also compared AD GWAS results from the catalog to GWAS results for 82 cardiac magnetic resonance imaging traits in UKB. These traits were derived through previous research and were returned to UKB in the category “Cardiac and aortic function #1.” In order to minimize the effects of population stratification, the cohort used for GWAS analysis for these traits was limited to individuals of White British ancestry and filtered to exclude participants with varying genetic and reported sex, sex chromosome aneuploidy, and relatives. After filtering, 26 335 participants remained in the discovery data set. We adjusted for the same covariates considered in previous research by Zhao et al.^30^

All GWASs used GRCh38 genome build.^31^ We filtered for SNVs with *P*<5 × 10^−8^ and identified colocalizations as SNV pairs located within 50 kb on the same chromosome. We then used the National Human Genome Research Institute‐European Bioinformatics Institute catalog to search for genes containing these SNVs.

## Results

### Demographic Characteristics

In both UKB and AoU, participants with AD were generally older, more likely to have diabetes, and had lower educational attainment and income compared with those without AD. Cases with AD were also more likely to be former or current smokers and less likely to report frequent alcohol consumption. Body mass index differences were minimal between groups. Racial and ethnic representation was broader in AoU, whereas UKB was predominantly White (94.1%). These findings are summarized in Tables 1 and 2.

**Table 1:** Demographic Information About UKB Full Cohort and Split by AD Status

Covariate	Status	Full cohort	Without AD	With AD
Diabetes	No	457 702 (91.2%)	454 399 (91.2%)	3303 (80.0%)
	Yes	44 431 (8.8%)	43 605 (8.8%)	826 (20.0%)
Age, y		56.53 (8.09)	56.46 (8.08)	64.65 (4.24)
Sex	Female	273 158 (54.4%)	271 004 (54.4%)	2154 (52.2%)
	Male	228 975 (45.6%)	227 000 (45.6%)	1975 (47.8%)
Age completed full‐time education, y		16.36 (2.83)	16.36 (3.45)	15.71 (3.54)
	NA	165 587 (33.0%)	164 722 (33.1%)	865 (20.9%)
Body mass index, kg/m^2^		27.43 (4.80)	27.43 (4.80)	27.47 (4.69)
	NA	3103 (0.6%)	3063 (0.6%)	40 (1.0%)
Smoking status	Never	273 328 (54.4%)	271 336 (54.5%)	1992 (48.2%)
	Previous	172 920 (34.4%)	171 206 (34.4%)	1714 (41.5%)
	Current	52 937 (10.5%)	52 566 (10.6%)	371 (9.0%)
	Prefer not to answer	2948 (0.6%)	2896 (0.6%)	52 (1.3%)
Depression/bipolar disorder	No	468 716 (93.3%)	464 772 (93.3%)	3944 (95.5%)
	Yes	33 417 (6.7%)	33 232 (6.7%)	185 (4.5%)
D of moderate physical activity each wk		3.63 (2.33)	3.62 (2.33)	3.95 (2.38)
	Missing	27 251 (5.4%)	26 876 (5.4%)	375 (9.1%)
D of vigorous physical activity each wk		1.84 (1.96)	1.84 (1.96)	1.85 (2.12)
	Missing	27 565 (5.5%)	27 157 (5.5%)	408 (9.9%)
Alcohol consumption	Daily or almost daily	603 (0.1%)	595 (0.1%)	8 (0.2%)
	3–4 times a wk	101 716 (20.3%)	100 933 (20.3%)	783 (19.0%)
	1–2 times a wk	115 357 (23.0%)	114 552 (23.0%)	805 (19.5%)
	1–3 times a mo	129 186 (25.7%)	128 215 (25.7%)	971 (23.5%)
	Special occasions only	55 811 (11.1%)	55 392 (11.1%)	419 (10.1%)
	Never	57 967 (11.5%)	57 379 (11.5%)	588 (14.2%)
	Prefer not to answer	41 493 (8.3%)	40 938 (8.2%)	555 (13.4%)
Annual household income	<£18 000	49 778 (9.9%)	49 203 (9.9%)	575 (13.9%)
	£18 000–£30 999	21 295 (4.2%)	20 900 (4.2%)	395 (9.6%)
	£31 000–£51 999	97 143 (19.3%)	95 768 (19.2%)	1375 (33.3%)
	£52 000–£100 000	108 101 (21.5%)	107 135 (21.5%)	966 (23.4%)
	>£100 000	110 690 (22.0%)	110 226 (22.1%)	464 (11.2%)
	Do not know	86 204 (17.2%)	86 009 (17.3%)	195 (4.7%)
	Prefer not to answer	28 922 (5.8%)	28 763 (5.8%)	159 (3.9%)
Racial or ethnic background	White	472 365 (94.1%)	468 415 (94.1%)	3950 (95.7%)
	Black	8048 (1.6%)	7990 (1.6%)	58 (1.4%)
	Asian	9872 (2.0%)	9818 (2.0%)	54 (1.3%)
	Chinese	1571 (0.3%)	1567 (0.3%)	4 (0.1%)
	Mixed	2950 (0.6%)	2937 (0.6%)	13 (0.3%)
	Other*	4552 (0.9%)	4529 (0.9%)	23 (0.6%)
	Unknown	217 (0.0%)	216 (0.0%)	1 (0.0%)
	Prefer not to answer	2558 (0.5%)	2532 (0.5%)	26 (0.6%)

AD indicates Alzheimer's disease; and UKB, UK Biobank.

* Indicates self‐reported value of race.

**Table 2:** Demographic Information About AoU Full Cohort and Split by AD Status*

Covariate	Status	Full cohort	Without AD	With AD
Diabetes	No	237 281 (82.7%)	236 807 (82.7%)	474 (61.2%)
	Yes	49 730 (17.3%)	49 430 (17.3%)	300 (38.8%)
Age		58.01 (16.96)	57.95 (16.93)	79.95 (9.88)
Sex	Female	172 401 (60.1%)	171 999 (60.1%)	402 (51.9%)
	Male	108 739 (37.9%)	108 388 (37.9%)	351 (45.3%)
	Other/prefer not to answer	5871 (2.0%)	5850 (1.9%)	21 (2.7%)
Highest level of education	Grade 4 or lower	2969 (1.0%)	2948 (1.0%)	21 (2.7%)
	Grades 5–8	6745 (2.4%)	6700 (2.3%)	45 (5.8%)
	Grades 9–11	18 153 (6.3%)	18 115 (6.3%)	38 (4.9%)
	Grade 12 or GED	56 778 (19.8%)	56 647 (19.8%)	131 (16.9%)
	1–3 years college	72 938 (25.4%)	72 780 (25.4%)	158 (20.4%)
	College graduate	62 550 (21.8%)	62 394 (21.8%)	156 (20.2%)
	Advanced degree	57 696 (20.1%)	57 494 (20.1%)	202 (26.1%)
	Prefer not to answer	9182 (3.2%)	9159 (3.2%)	23 (3.0%)
BMI		29.90 (7.64)	29.90 (7.64)	28.65 (6.57)
	Missing	12 715 (4.4%)	12 676 (4.4%)	39 (5.0%)
Smoked 100 cigarettes in lifetime	No	164 021 (57.1%)	163 612 (57.2%)	409 (52.8%)
	Yes	114 595 (39.9%)	114 253 (39.9%)	342 (44.2%)
	Don’t know/prefer not to answer	8395 (3.0%)	8372 (3.0%)	23 (3.0%)
Depression	No	208 532 (72.7%)	208 193 (72.7%)	339 (43.8%)
	Yes	78 479 (27.3%)	78 044 (27.3%)	435 (56.2%)
Frequency of alcoholic drinks over past year	4 or More Per Week	30 087 (10.5%)	30 003 (10.5%)	84 (10.9%)
	2 to 3 per wk	34 981 (12.2%)	34 920 (12.2%)	61 (7.9%)
	2 to 4 per mo	52 212 (18.2%)	52 117 (18.2%)	95 (12.3%)
	Monthly or less	84 407 (29.4%)	84 226 (29.4%)	181 (23.4%)
	Never	46 653 (16.3%)	46 422 (16.2%)	231 (29.8%)
	Prefer not to answer	38 671 (13.5%)	38 549 (13.5%)	122 (15.8%)
Annual household income	<$10,000	40 856 (14.2%)	40 781 (14.2%)	75 (9.7%)
	$10,000–$25,000	33 965 (11.8%)	33 851 (11.8%)	114 (14.7%)
	$25,000–$35,000	20 267 (7.1%)	20 216 (7.1%)	51 (6.6%)
	$35,000–$50,000	22 252 (7.8%)	22 184 (7.8%)	68 (8.8%)
	$50,000–$75,000	29 186 (10.2%)	29 106 (10.2%)	80 (10.3%)
	$75,000–$100,000	22 359 (7.8%)	22 308 (7.8%)	51 (6.6%)
	$100,000–$150,000	27 202 (9.5%)	27 146 (9.5%)	56 (7.2%)
	$150,000–$200,000	12 496 (4.4%)	12 472 (4.4%)	24 (3.1%)
	>$200,000	17 507 (6.1%)	17 480 (6.1%)	27 (3.5%)
	Prefer not to answer	60 921 (21.2%)	60 693 (21.2%)	228 (29.5%)
Race or ethnicity	White	150 402 (52.4%)	149 897 (52.4%)	505 (65.2%)
	Black	57 177 (19.9%)	57 088 (19.9%)	89 (11.5%)
	Hispanic	47 699 (16.6%)	47 579 (16.6%)	120 (15.5%)
	Asian	8038 (2.8%)		
	Other/unknown	23 695 (8.3%)	23 648 (8.3%)	47 (6.1%)

AD indicates Alzheimer's disease; AoU, All of Us; BMI, body mass index; and GED, General Educational Development.

* Asian AD case distribution is suppressed to protect participant privacy.

### Cardiovascular Disease Subtypes and AD Prevalence

CVD subtypes showed varying prevalence across cohorts. In UKB, hypertension was most common (32.3%), followed by chronic ischemic heart disease and cardiac arrhythmias. In AoU, there was a slightly higher prevalence of hypertension (36.6%), which was followed by chronic ischemic heart disease and cardiac arrhythmias. AD prevalence was higher among individuals with each CVD subtype compared with those without, with the strongest differences seen in hypotension, heart failure, cardiac arrhythmias, and cerebral infarction (Tables 3 and 4). AD rates were significantly different in groups with and without each form of CVD for all groups except pulmonary embolism in AoU, which was removed from further analysis due to the existence of only 2 cases with AD.

**Table 3:** Counts of UKB Participants With Each Subtype of CVD Overall and Split by AD Status

CVD subtype	Prevalence	AD with CVD	AD without CVD	*P* value
Hypertension	162 261 (32.3%)	2462 (1.5%)	1667 (0.5%)	1.84×10^−310^
Chronic ischemic heart disease	53 430 (10.6%)	934 (1.7%)	3195 (0.7%)	2.11×10^−138^
Cardiac arrhythmia	49 862 (9.9%)	1055 (2.1%)	3074 (0.7%)	1.30×10^−248^
Angina pectoris	34 167 (6.8%)	636 (1.9%)	3493 (0.7%)	2.78×10^−107^
Hypotension	21 310 (4.2%)	746 (3.5%)	3383 (0.7%)	0
Heart failure	20 075 (4%)	468 (2.3%)	3661 (0.8%)	1.44×10^−128^
Acute myocardial infarction	17 822 (3.5%)	251 (1.4%)	3878 (0.8%)	1.64×10^−18^
Chronic rheumatic heart disease	11 284 (2.2%)	210 (1.9%)	3919 (0.8%)	8.45×10^−35^
Cerebral infarction	10 345 (2.1%)	241 (2.3%)	3888 (0.8%)	1.5×10^−65^
Pulmonary embolism	10 067 (2%)	174 (1.7%)	3955 (0.8%)	4.78×10^−24^

AD indicates Alzheimer's disease; CVD, cardiovascular disease; and UKB, UK Biobank.

**Table 4:** Counts of AoU Participants With Each Subtype of CVD Overall and Split by AD Status

CVD subtype	Prevalence	AD with CVD	AD without CVD	*P* value
Hypertension	104 997 (36.6%)	599 (0.6%)	175 (0.1%)	8.83×10^−123^
Chronic ischemic heart disease	32 160 (11.2%)	323 (1%)	451 (0.2%)	2×10^−159^
Cardiac arrhythmia	21 686 (7.6%)	234 (1.1%)	540 (0.2%)	1.78×10^−125^
Heart failure	18 604 (6.5%)	195 (1%)	579 (0.2%)	8.05×10^−99^
Hypotension	17 473 (6.1%)	192 (1.1%)	582 (0.2%)	9.92×10^−105^
Angina pectoris	8990 (3.1%)	75 (0.8%)	699 (0.3%)	2.92×10^−25^
Chronic rheumatic heart disease	8035 (2.8%)	75 (0.9%)	699 (0.3%)	9.6×10^−31^
Cerebral infarction	7927 (2.8%)	107 (1.3%)	667 (0.2%)	5.38×10^−78^
Acute myocardial infarction	6580 (2.3%)	54 (0.8%)	720 (0.3%)	8.07×10^−18^

AD indicates Alzheimer's disease; AoU: All of Us; and CVD, cardiovascular disease.

### Associations Between CVD Subtypes and AD

**Figure 1:**
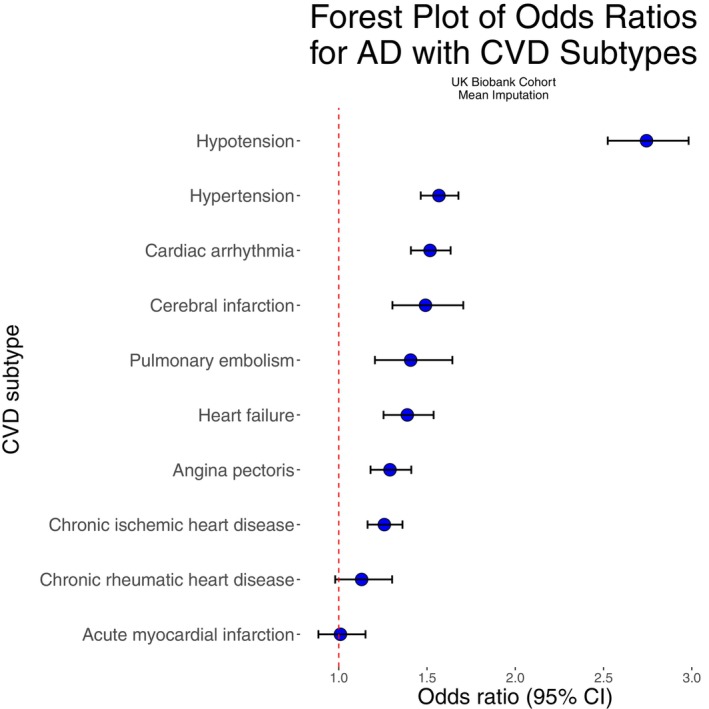
Forest plot of odds ratios for AD with CVD subtypes in the UK Biobank cohort. Blue dots represent odds ratio point estimates, and error bars show the 95% CI. The dotted red line shows the significance threshold. By far the strongest association was noted between hypotension and AD. All CVD subtypes besides acute myocardial infarction and chronic rheumatic heart disease had significant relationships with AD. AD indicates Alzheimer's disease; and CVD, cardiovascular disease.

**Figure 2:**
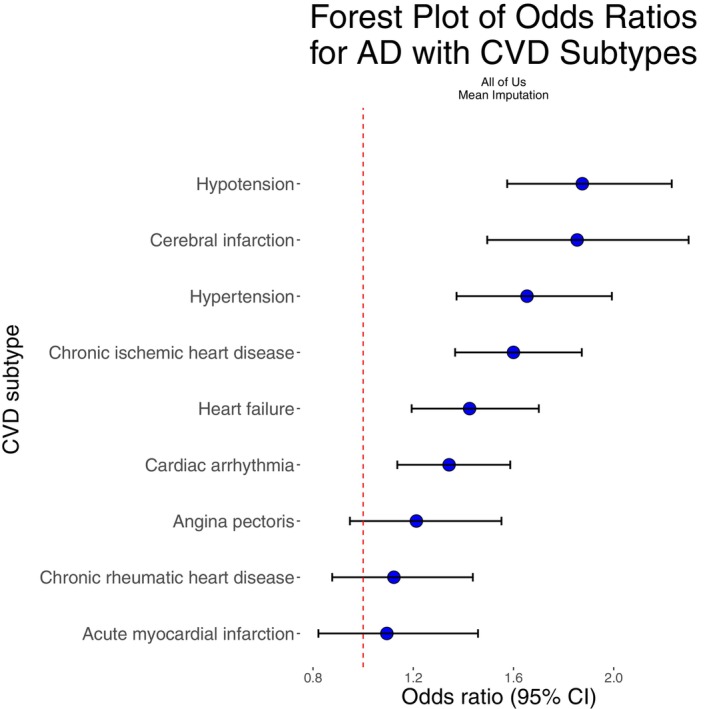
Forest plot of odds ratios for AD with CVD subtypes in the All of Us cohort. Blue dots represent odds ratio point estimates, and error bars show the 95% CI. The dotted red line shows the significance threshold. Note the strongest associations between AD and hypotension, cerebral infarction, and hypertension. All CVD subtypes were significant at the 95% confidence level except for acute myocardial infarction, chronic rheumatic heart disease, and angina pectoris. AD indicates Alzheimer's disease; and CVD, cardiovascular disease.

Logistic regression analysis adjusted for relevant covariates revealed that nearly all CVD subtypes were significantly associated with higher odds of AD in both cohorts (Figures 1 and 2). Covariate adjustment consistently shrunk OR estimates, highlighting the value of accounting for these variables in reducing confounding (Tables S1–S4). In UKB, the strongest association was observed between hypotension and AD (OR, 2.74 [95% CI, 2.52–2.98]), followed by hypertension (OR, 1.57 [95% CI, 1.46–1.68]), cardiac arrhythmias (OR, 1.52 [95% CI, 1.41–1.63]), and cerebral infarction (OR, 1.49 [95% CI, 1.30–1.71]). AMI (OR, 1.01 [95% CI, 0.88–1.15]) and chronic rheumatic heart disease (OR, 1.13 [95% CI, 0.98–1.30]) were not significantly associated with AD.

Findings in AoU generally mirrored those from UKB. Again, hypotension showed the strongest association with AD (OR, 1.97 [95% CI, 1.57–2.23]), followed by cerebral infarction (OR, 1.85 [95% CI, 1.50–2.30]) and hypertension (OR, 1.65 [95% CI, 1.37–1.99]). Angina pectoris (OR, 1.21 [95% CI, 0.95–1.55]), chronic rheumatic heart disease (OR, 1.12 [95% CI, 0.88–1.44]), and AMI (OR, 1.09 [95% CI, 0.82–1.46]) were not significantly associated. OR estimates in AoU had wider CIs, indicating the greater variance of estimates due to the smaller proportion of CVD events in the AD population. This variance may also reflect the greater racial and ethnic diversity, broader socioeconomic representation, and the lack of adjustment for physical activity in AoU.

OR calculations were nearly identical when the multiple imputation by chained equations method was used for imputation, a sensitivity analysis that validated our use of imputed means. ORs also remained stable when performing Firth regression to assess the effects of the case–control imbalance. Sensitivity analysis results can be found in Figures S1 and S2, Tables S5–S8.

**Figure 3:**
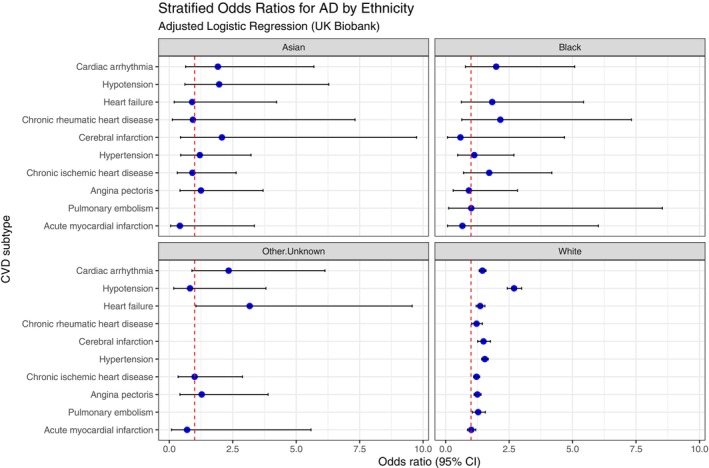
Forest plot of racially and ethnically stratified odds ratios for AD with CVD subtypes in the UKB cohort. Due to limited case counts across minority strata, odds ratio estimates were highly unstable in the UKB. Results of CVD subtypes for which upper bound of the odds ratio CI was >10 are suppressed. AD indicates Alzheimer's disease; CVD, cardiovascular disease; and UKB, UK Biobank.

**Figure 4:**
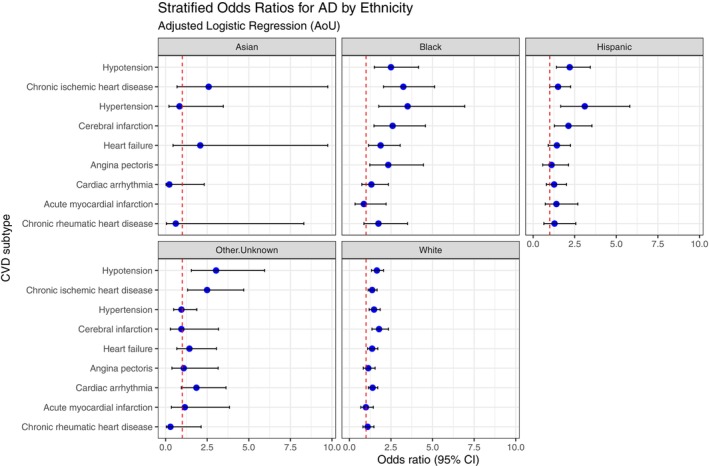
Forest plot of racially and ethnically stratified odds ratios for AD with CVD subtypes in the AoU cohort. Results of CVD subtypes for which upper bound of the odds ratio CI was >10 are suppressed. Hypertension and hypotension exhibited the strongest associations with AD across strata, with hypertension having a larger odds ratio in Black and Hispanic populations. Note the generally stronger associations between CVD and AD in Black and Hispanic minority groups than the White population. AD indicates Alzheimer's disease; AoU, All of Us; and CVD, cardiovascular disease.

Stratified sampling indicated generally stronger relationships between AD and CVD for Black and Hispanic populations than the White population (Figures 3 and 4, Tables S9 and S10). In the UKB, OR estimates were highly unstable in minority populations due to small sample size. AoU is more diverse, so we were able to obtain more informative OR estimates in minority populations. Notably, hypotension had the strongest relationship with AD in the White cohort, whereas hypertension had a stronger relationship in the Black and Hispanic subgroups.

**Figure 5:**
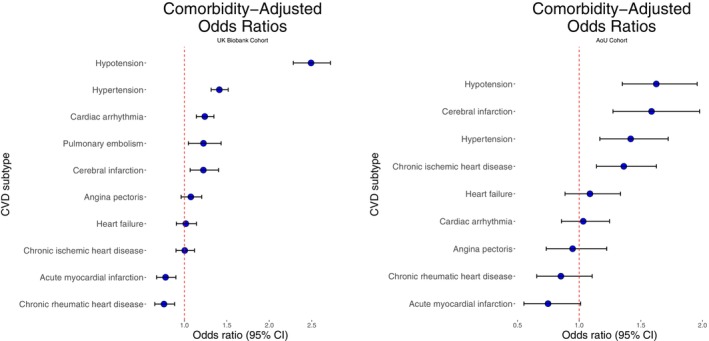
UK Biobank and All of Us CVD and AD odds ratios with comorbidity adjustment. For both data sets, the odds ratio ranks were preserved, but the effect size decreased when adjusting for comorbidities. AD indicates Alzheimer's disease; AoU, All of Us; and CVD, cardiovascular disease.

When performing logistic regression with adjustment for cardiovascular comorbidities, we found CVD subtype–AD relationships to follow the same rank as unadjusted logistic regression (Figure 5, Tables S11 and S12). Effect sizes generally shrunk. Some CVD subtypes, namely chronic rheumatic heart disease and AMI, had negative associations with AD after comorbidity adjustment.

**Figure 6:**
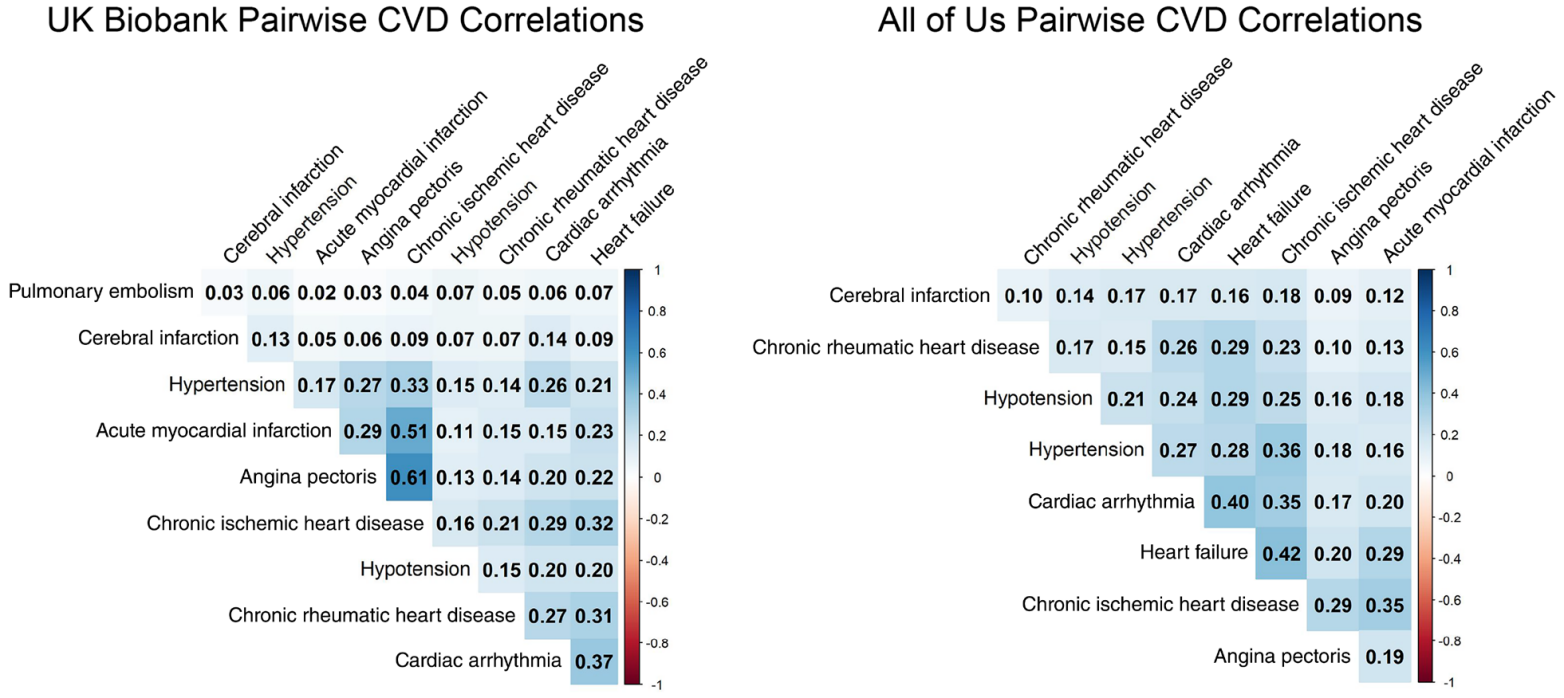
Pairwise correlations between CVD subtypes in UKB and AoU. In both UKB and AoU, correlations between CVD subtypes were moderate, with the strongest pairwise correlation found between angina pectoris and chronic ischemic heart disease (*ρ*=0.61). AD indicates Alzheimer's disease; AoU, All of Us; and CVD, cardiovascular disease.

We calculated pairwise correlation coefficients between CVD subtypes to determine whether comorbidity‐adjusted ORs were unstable because of multicollinearity (Figure 6). In UKB, the strongest correlation was between angina pectoris and chronic ischemic heart disease (*ρ*=0.61).

### Genetic Overlap Between CVD and AD

To explore shared genetic architecture, we identified SNVs significantly associated with both CVD‐ and AD‐related traits that were located within 50 kb of one another (*P*<5 × 10^−8^). A total of 164 unique SNV pairs were found to be proximal across data sets, with the strongest overlaps observed between AD and traits such as angina pectoris, left ventricular myocardial wall thickness, and coronary artery disease.

Several loci stood out for high overlap, including:

- **19q13.32**, encompassing *APOE*, *TOMM40*, *APOC1*, *APOC1P1*, *NECTIN2*, and *APOC4‐APOC2*, linked to lipid metabolism and both AD and cardiovascular traits.^32^, ^33^, ^34^, ^35^
- **17q21.31**, home to *MAPT*, *KANSL1*, and *WNT3* with links to ventricular wall thickness and AD risk.^30^
- **11p11.2**, containing *PSMC3*, *SPI1*, and *RAPSN*, genes implicated in neuroinflammation, immune response, and cardiac structure.^36^, ^37^

A table of proximal SNVs and their related heart and brain traits can be viewed in Table S13.

## DISCUSSION

This study used 2 large, demographically distinct biobank data sets—UKB and AoU—to investigate associations between AD and multiple CVD subtypes. Our findings demonstrate that most CVD subtypes are significantly associated with higher odds of AD, with hypotension emerging as the strongest and most consistent correlation in both cohorts. These results highlight the importance of examining the heart–brain axis beyond general CVD risk and underscore the value of differentiating CVD subtypes in dementia research.

Notably, hypotension showed the highest odds of AD across both data sets. We defined hypotension with the *ICD‐10* code I95, which encompasses diverse subtypes, including idiopathic hypotension, orthostatic hypotension, hypotension due to drugs, and other hypotension.^38^ Although these different forms of hypotension may have different relationships with AD, they were grouped together to ensure sufficient sample size for statistical analysis. Although hypertension has been extensively studied as a modifiable AD risk factor, hypotension is comparatively understudied despite its high prevalence among older adults.^39^ Both chronic and orthostatic hypotension have been associated with cerebral hypoperfusion, oxidative stress, and tau pathology—mechanisms that could exacerbate or accelerate AD progression.^2^, ^13^ Conversely, AD‐related dysfunction of the autonomic nervous system may impair cardiovascular regulation, suggesting a bidirectional relationship.^40^

The relationships between hypotension, hypertension, and AD were found to differ by ethnic strata. Hypotension was more strongly associated with AD for White individuals, whereas hypertension had the strongest relationship in Black and Hispanic populations. This pattern may be explained by greater rates of uncontrolled hypertension in Black and Hispanic populations.^41^ These populations tend to face greater socioeconomic barriers to treatment, an underlying factor that may explain detrimental effects on cognition due to hypertension or facilitate the progression of AD and hypertension in tandem.^42^

Hypertension and cerebral infarction also demonstrated strong associations with AD, consistent with established literature linking these conditions to vascular pathology,^2^, ^4^, ^15^, ^43^, ^44^ white matter damage,^7^ and cognitive decline.^5^, ^45^ Cardiac arrhythmias, known to increase stroke risk and independently linked to cognitive impairment, were similarly associated with AD.^16^, ^46^ These findings support a broader vascular contribution to AD and indicate that multiple CVD pathways, particularly those impacting cerebral blood flow, may converge in AD pathophysiology.

Notably, AMI was not significantly associated with AD. This aligns with prior research suggesting that although AMI may contribute to cognitive decline through systemic inflammation or hypoxia,^47^ its direct link to AD pathology remains limited.^14^, ^48^ However, AMI's indirect effects on brain health warrant further investigation, particularly regarding rates of long‐term cognitive decline post infarction.^47^, ^49^, ^50^ The other CVD subtype not significantly associated with AD in our analysis was chronic rheumatic heart disease. Rheumatic heart disease is often an early‐stage form of CVD that later progresses to other subtypes, particularly cardiac arrhythmias.^51^ Rheumatic heart disease may contribute to AD risk only after the development of further comorbidities.

After performing comorbidity adjustment, hypotension, hypertension, and cerebral infarction remained significantly associated with AD in both cohorts. In UKB, cardiac arrhythmia and pulmonary embolism were significantly linked after adjustment, and chronic ischemic heart disease was in AoU. Surprisingly, AMI and chronic rheumatic heart disease became negatively associated with AD after comorbidity adjustment, a finding that is inconsistent with existing literature.^47^ This unexpected inverse association likely reflects model instability due to shared variance among CVD subtypes, residual confounding, or selection effects rather than a protective relationship.

Our genetic proximity analysis identified overlapping SNVs between CVD and AD traits, especially in loci involving *APOE*, *MAPT*, *SPI1*, and *WNT3*. The *APOE* region (19q13.32) remains the most prominent shared locus, given its well‐established roles in lipid metabolism,^32^, ^34^ blood–brain barrier integrity,^6^, ^33^ and both cardiovascular and neurodegenerative diseases.^32^, ^33^, ^35^ Interestingly, we also observed overlap between myocardial wall thickness traits and AD‐related SNVs in several regions, including 17q21.31 (*MAPT*) and 11p11.2 (*PSMC3*, *RAPSN*), pointing to potential shared mechanisms involving cardiac remodeling and brain structure integrity.^30^, ^52^, ^53^

Our genetic analysis used a relatively simple approach, but other studies of similar populations have used more advanced colocalization methods in similar populations. In a landmark study, Zhao et al. performed colocalization analysis between markers of cardiac structure and function and neurological and psychological traits with the UKB data set.^30^ They found colocalizations between heart and brain magnetic resonance imaging‐derived traits in locus 19q13.32. Locus 17q21.31 was associated with cognitive ability, dementia, and cardiac wall thickness traits. Locus 11p11.2 was associated with cardiac wall thickness, mental health‐related traits, and cognitive ability. Another study using 7 GWASs of European ancestry identified colocalization between AD and diastolic blood pressure in locus 11p11.2 surrounding *SPI1*.^54^ These findings corroborate the importance of the loci we found to be important for heart and brain health.

Differences between the 2 cohorts—UKB's healthier, less diverse sample versus AoU's more representative and clinically diverse population—underscore the generalizability of our findings. The replicated associations across these distinct cohorts strengthen the robustness of our results, and our ethnically stratified associations highlight the need for tailored prevention strategies that address specific CVD subtypes in diverse populations. The statistical validity of our results was confirmed through sensitivity analysis with multiple imputation by chained equations imputation and Firth regression. Additionally, we performed logistic regression with adjustment for cardiovascular comorbidities, providing insight into the multivariate relationships between CVD subtypes and AD.

Despite the strengths of large‐scale, multicohort replication and integration of genetic data, this study has limitations. First, its cross‐sectional design precludes causal inference, and the directionality of associations cannot be determined. Second, AD and CVD diagnoses were based on *ICD‐10* codes, which may underrepresent true disease prevalence due to undiagnosed or misclassified cases. The direction of bias this imprecision may introduce is uncertain. On one hand, underreported diagnoses may mean that we missed some individuals with both CVD and AD. Conversely, subjects with CVD but not AD, or AD but not CVD, may have been missed. Third, although we adjusted for cardiovascular comorbidities, the challenges of multicollinearity in regression made it difficult to differentiate the effects of distinct CVD subtypes.^55^, ^56^ Additionally, our genetic analysis, although informative, used proximity‐based colocalization rather than formal methods such as Mendelian randomization or transcriptome‐wide association studies, which could better infer causality or gene expression effects.

## Conclusions

In this study, we investigated the relationship between AD and multiple CVD subtypes across 2 large, diverse biobanks: the UK Biobank and All of Us. We found that most CVD subtypes were significantly associated with AD, with hypotension showing the strongest association in both data sets. Other conditions like hypertension, cerebral infarction, and cardiac arrhythmias also demonstrated consistent positive associations, reinforcing the central role of vascular health in cognitive decline. AMI was not significantly linked to AD, consistent with prior literature.

Genetic analyses revealed that several AD‐ and CVD‐associated SNVs were located near each other, especially in regions containing *APOE*, *MAPT*, and genes involved in myocardial structure. These results should be interpreted as hypothesis generating rather than evidence of causal genetic mechanisms, but they suggest a potentially shared genetic basis between heart and brain pathology. Although the cross‐sectional design and reliance on *ICD‐10* codes limit causal inference, our findings underscore the importance of cardiovascular health in AD risk and highlight specific CVD subtypes that may warrant increased attention in dementia risk stratification and prevention strategies.

## Sources of Funding

This research has been conducted using the UK Biobank Resource under application number 61915. It was in part supported by grants from the National Institutes of Health, USA (1R15HL172198,1R15HL173852, and U19AG055373), American Heart Association (25AIREA1377168), and a Michigan Technological University Undergraduate Research Internship Program.

## Disclosures

None.

## Supporting information

Tables S1–S13
Figures S1–S2

STROBEchecklist
