# Supplementary material for: Cardiovascular Disease Subtypes and Alzheimer's Disease: Phenotypic and Genetic Associations in the UK Biobank and All of Us Research Program
Source: J Am Heart Assoc. 2026 Jun 10;15(12):e046172. doi: 10.1161/JAHA.125.046172 (PMC13323167; doi:10.1161/JAHA.125.046172)
Supplement: Supplementary file 1 — Tables S1–S13 Figures S1–S2 [file JAH3-15-e046172-s002.pdf]

SUPPLEMENTAL MATERIAL

Odds Ratios and Confidence Intervals

Table S1 - UKB CVD-AD mean-imputed odds ratio estimates before covariate adjustment with 95% confidence intervals. (Abbreviations: UKB: UK Biobank, AD: Alzheimer's disease, CVD: Cardiovascular disease, OR: Odds ratio)

CVD Subtype	OR	Lower Bound	Upper Bound
Hypotension	5.12	4.72	5.55
Cardiac.Arrhythmia	3.16	2.94	3.39
Hypertension	3.13	2.94	3.33
Heart.Failure	3.12	2.83	3.44
Cerebral.Infarction	2.99	2.62	3.41
Angina.Pectoris	2.52	2.32	2.75
Chronic.Ischemic.Heart.Disease	2.48	2.31	2.67
Chronic.Rheumatic.Heart.Disease	2.36	2.05	2.71
Pulmonary.Embolism	2.17	1.86	2.53
Acute.Myocardial.Infarction	1.77	1.56	2.01

Table S2 - AoU CVD-AD mean-imputed odds ratio estimates before covariate adjustment with 95% confidence intervals. (Abbreviations: AoU: All of Us, AD: Alzheimer's disease, CVD: Cardiovascular disease, OR: Odds ratio)

CVD Subtype	OR	Lower Bound	Upper Bound
Hypertension	5.96	5.04	7.06
Chronic.Ischemic.Heart.Disease	5.72	4.96	6.61
Cerebral.Infarction	5.71	4.65	7.01
Cardiac.Arrhythmia	5.35	4.58	6.24
Hypotension	5.13	4.36	6.05
Heart.Failure	4.90	4.16	5.77
Chronic.Rheumatic.Heart.Disease	3.75	2.95	4.76
Angina.Pectoris	3.34	2.63	4.24
Acute.Myocardial.Infarction	3.21	2.44	4.24

Table S3 - UKB CVD-AD mean-imputed and covariate-adjusted odds ratio estimates with 95% confidence intervals. (Abbreviations: UKB: UK Biobank, AD: Alzheimer's disease, CVD: Cardiovascular disease, OR: Odds ratio)

CVD Subtype	OR	Lower Bound	Upper Bound
Hypotension	2.74	2.52	2.98
Hypertension	1.57	1.46	1.68
Cardiac.Arrhythmia	1.52	1.41	1.63
Cerebral.Infarction	1.49	1.30	1.71
Pulmonary.Embolism	1.41	1.20	1.64

Heart.Failure	1.39	1.25	1.54
Angina.Pectoris	1.29	1.18	1.41
Chronic.Ischemic.Heart.Disease	1.26	1.16	1.36
Chronic.Rheumatic.Heart.Disease	1.13	0.98	1.30
Acute.Myocardial.Infarction	1.01	0.88	1.15

Table S4 - AoU CVD-AD mean-imputed and covariate-adjusted odds ratio estimates with 95% confidence intervals. (Abbreviations: AoU: All of Us, AD: Alzheimer's disease, CVD: Cardiovascular disease, OR: Odds ratio)

CVD Subtype	OR	Lower Bound	Upper Bound
Hypotension	1.87	1.57	2.23
Cerebral.Infarction	1.85	1.50	2.30
Hypertension	1.65	1.37	1.99
Chronic.Ischemic.Heart.Disease	1.60	1.37	1.87
Heart.Failure	1.42	1.19	1.70
Cardiac.Arrhythmia	1.34	1.14	1.59
Angina.Pectoris	1.21	0.95	1.55
Chronic.Rheumatic.Heart.Disease	1.12	0.88	1.44
Acute.Myocardial.Infarction	1.09	0.82	1.46

Table S5 - UKB MICE-imputed CVD-AD odds ratio estimates with 95% confidence intervals. (Abbreviations: UKB: UK Biobank, MICE: Multiple imputation by chained equations, CVD: Cardiovascular disease, AD: Alzheimer's disease, OR: Odds ratio)

CVD Subtype	OR	Lower Bound	Upper Bound
Hypotension	2.74	2.53	2.98
Hypertension	1.57	1.47	1.68
Cardiac.Arrhythmia	1.52	1.41	1.64
Cerebral.Infarction	1.49	1.30	1.71
Pulmonary.Embolism	1.41	1.21	1.65
Heart.Failure	1.39	1.26	1.54
Angina.Pectoris	1.29	1.18	1.41
Chronic.Ischemic.Heart.Disease	1.26	1.16	1.36
Chronic.Rheumatic.Heart.Disease	1.13	0.98	1.30
Acute.Myocardial.Infarction	1.01	0.89	1.15

Table S6 - AoU MICE-imputed CVD-AD odds ratio estimates with 95% confidence intervals. (Abbreviations: AoU: All of Us, MICE: Multiple imputation by chained equations, CVD: Cardiovascular disease, AD: Alzheimer's disease, OR: Odds ratio)

CVD Subtype	OR	Lower Bound	Upper Bound

Hypotension	1.87	1.57	2.23
Cerebral.Infarction	1.85	1.49	2.30
Hypertension	1.66	1.38	2.00
Chronic.Ischemic.Heart.Disease	1.60	1.37	1.87
Heart.Failure	1.43	1.20	1.71
Cardiac.Arrhythmia	1.34	1.14	1.59
Angina.Pectoris	1.21	0.95	1.55
Chronic.Rheumatic.Heart.Disease	1.12	0.88	1.44
Acute.Myocardial.Infarction	1.09	0.82	1.46

Table S7 - UKB CVD–AD Firth regression odds ratio estimates with 95% confidence intervals. (Abbreviations: UKB: UK Biobank, CVD: Cardiovascular disease, AD: Alzheimer's disease, OR: Odds ratio)

CVD Subtype	OR	Lower Bound	Upper Bound
Hypotension	2.74	2.52	2.98
Hypertension	1.57	1.46	1.68
Cardiac.Arrhythmia	1.52	1.41	1.63
Cerebral.Infarction	1.49	1.30	1.70
Pulmonary.Embolism	1.41	1.20	1.64
Heart.Failure	1.39	1.25	1.54
Angina.Pectoris	1.29	1.18	1.41
Chronic.Ischemic.Heart.Disease	1.26	1.16	1.36
Chronic.Rheumatic.Heart.Disease	1.13	0.98	1.30
Acute.Myocardial.Infarction	1.01	0.88	1.15

Table S8 - AoU CVD–AD Firth regression odds ratio estimates with 95% confidence intervals. (Abbreviations: AoU: All of Us, CVD: Cardiovascular disease, AD: Alzheimer's disease, OR: Odds ratio)

CVD Subtype	OR	Lower Bound	Upper Bound
Hypotension	1.88	1.57	2.23
Cerebral.Infarction	1.86	1.49	2.29
Hypertension	1.65	1.37	1.99
Chronic.Ischemic.Heart.Disease	1.60	1.37	1.87
Heart.Failure	1.43	1.19	1.70
Cardiac.Arrhythmia	1.34	1.14	1.59
Angina.Pectoris	1.22	0.95	1.55
Chronic.Rheumatic.Heart.Disease	1.13	0.88	1.43
Acute.Myocardial.Infarction	1.10	0.82	1.45

Table S9 - UKB ethnically stratified CVD-AD odds ratio estimates with 95% confidence intervals. (Abbreviations: UKB: UK Biobank, CVD: Cardiovascular disease, AD: Alzheimer's disease, OR: Odds ratio)

CVD Subtype	Ethnicity	OR	Lower Bound	Upper Bound
Pulmonary.Embolism	Asian	6.60	0.77	56.78
Cerebral.Infarction	Asian	2.07	0.44	9.75
Hypotension	Asian	1.96	0.61	6.28
Cardiac.Arrhythmia	Asian	1.91	0.64	5.70
Angina.Pectoris	Asian	1.25	0.42	3.70
Hypertension	Asian	1.20	0.45	3.22
Chronic.Rheumatic.Heart.Disease	Asian	0.93	0.12	7.32
Chronic.Ischemic.Heart.Disease	Asian	0.91	0.32	2.63
Heart.Failure	Asian	0.90	0.19	4.23
Acute.Myocardial.Infarction	Asian	0.42	0.05	3.35
Hypotension	Black	4.77	1.77	12.87
Chronic.Rheumatic.Heart.Disease	Black	2.15	0.63	7.32
Cardiac.Arrhythmia	Black	1.99	0.78	5.09
Heart.Failure	Black	1.83	0.62	5.43
Chronic.Ischemic.Heart.Disease	Black	1.72	0.70	4.18
Hypertension	Black	1.13	0.47	2.69
Pulmonary.Embolism	Black	1.01	0.12	8.54
Angina.Pectoris	Black	0.92	0.30	2.83
Acute.Myocardial.Infarction	Black	0.66	0.07	6.02
Cerebral.Infarction	Black	0.58	0.07	4.68
Hypertension	Other.Unknown	3.40	1.15	10.01
Heart.Failure	Other.Unknown	3.17	1.05	9.57
Cerebral.Infarction	Other.Unknown	3.14	0.78	12.59
Cardiac.Arrhythmia	Other.Unknown	2.34	0.89	6.13
Angina.Pectoris	Other.Unknown	1.28	0.42	3.89
Chronic.Ischemic.Heart.Disease	Other.Unknown	1.00	0.35	2.88
Hypotension	Other.Unknown	0.82	0.17	3.81
Acute.Myocardial.Infarction	Other.Unknown	0.70	0.09	5.59
Pulmonary.Embolism	Other.Unknown	0.00	0.00	Inf
Chronic.Rheumatic.Heart.Disease	Other.Unknown	0.00	0.00	Inf
Hypotension	White	2.69	2.43	2.99
Hypertension	White	1.54	1.42	1.68
Cerebral.Infarction	White	1.49	1.26	1.76
Cardiac.Arrhythmia	White	1.45	1.32	1.59
Heart.Failure	White	1.36	1.20	1.55
Pulmonary.Embolism	White	1.28	1.05	1.56
Angina.Pectoris	White	1.25	1.12	1.40

Chronic.Rheumatic.Heart.Disease	White	1.22	1.02	1.45
Chronic.Ischemic.Heart.Disease	White	1.21	1.10	1.34
Acute.Myocardial.Infarction	White	1.01	0.86	1.19

Table S10 - AoU ethnically stratified CVD-AD odds ratio estimates with 95% confidence intervals. (Abbreviations: AoU: All of Us, CVD: Cardiovascular disease, AD: Alzheimer's disease, OR: Odds ratio)

**CVD Subtype**	**Ethnicity**	**OR**	**Lower Bound**	**Upper Bound**
Acute.Myocardial.Infarction	Asian	3.50	0.54	22.79
Hypotension	Asian	3.10	0.63	15.31
Chronic.Ischemic.Heart.Disease	Asian	2.59	0.69	9.75
Heart.Failure	Asian	2.08	0.44	9.74
Cerebral.Infarction	Asian	0.98	0.09	10.68
Hypertension	Asian	0.83	0.20	3.47
Chronic.Rheumatic.Heart.Disease	Asian	0.61	0.04	8.31
Cardiac.Arrhythmia	Asian	0.22	0.02	2.32
Angina.Pectoris	Asian	0.00	0.00	Inf
Hypertension	Black	3.50	1.77	6.93
Chronic.Ischemic.Heart.Disease	Black	3.24	2.05	5.13
Cerebral.Infarction	Black	2.60	1.48	4.57
Hypotension	Black	2.50	1.50	4.16
Angina.Pectoris	Black	2.34	1.23	4.45
Heart.Failure	Black	1.88	1.15	3.05
Chronic.Rheumatic.Heart.Disease	Black	1.75	0.87	3.50
Cardiac.Arrhythmia	Black	1.32	0.75	2.34
Acute.Myocardial.Infarction	Black	0.87	0.34	2.21
Hypertension	Hispanic	3.10	1.66	5.81
Hypotension	Hispanic	2.20	1.41	3.44
Cerebral.Infarction	Hispanic	2.13	1.28	3.53
Chronic.Ischemic.Heart.Disease	Hispanic	1.50	0.99	2.25
Heart.Failure	Hispanic	1.43	0.91	2.25
Acute.Myocardial.Infarction	Hispanic	1.40	0.73	2.68
Chronic.Rheumatic.Heart.Disease	Hispanic	1.29	0.64	2.57
Cardiac.Arrhythmia	Hispanic	1.26	0.80	2.01
Angina.Pectoris	Hispanic	1.11	0.58	2.13
Hypotension	Other.Unknown	3.03	1.55	5.95
Chronic.Ischemic.Heart.Disease	Other.Unknown	2.49	1.32	4.70
Cardiac.Arrhythmia	Other.Unknown	1.85	0.94	3.63
Heart.Failure	Other.Unknown	1.43	0.67	3.05
Acute.Myocardial.Infarction	Other.Unknown	1.15	0.35	3.84

Angina.Pectoris	Other.Unknown	1.09	0.38	3.16
Hypertension	Other.Unknown	0.95	0.48	1.88
Cerebral.Infarction	Other.Unknown	0.95	0.28	3.18
Chronic.Rheumatic.Heart.Disease	Other.Unknown	0.29	0.04	2.13
Cerebral.Infarction	White	1.78	1.36	2.35
Hypotension	White	1.65	1.33	2.06
Hypertension	White	1.48	1.19	1.85
Cardiac.Arrhythmia	White	1.40	1.14	1.70
Chronic.Ischemic.Heart.Disease	White	1.37	1.13	1.67
Heart.Failure	White	1.37	1.10	1.71
Angina.Pectoris	White	1.14	0.83	1.55
Chronic.Rheumatic.Heart.Disease	White	1.10	0.82	1.48
Acute.Myocardial.Infarction	White	0.99	0.69	1.44

Table S11 - UKB comorbidity adjusted odds ratio estimates with 95% confidence intervals. (Abbreviations: UKB: UK Biobank, CVD: Cardiovascular disease, OR: Odds ratio)

CVD Subtype	OR	Lower Bound	Upper Bound
Hypotension	2.49	2.28	2.72
Hypertension	1.41	1.32	1.52
Cardiac.Arrhythmia	1.24	1.14	1.35
Pulmonary.Embolism	1.23	1.05	1.43
Cerebral.Infarction	1.22	1.07	1.40
Angina.Pectoris	1.08	0.96	1.20
Heart.Failure	1.02	0.91	1.14
Chronic.Ischemic.Heart.Disease	1.00	0.90	1.12
Acute.Myocardial.Infarction	0.78	0.67	0.90
Chronic.Rheumatic.Heart.Disease	0.76	0.65	0.89

Table S12 - AoU comorbidity adjusted CVD-AD odds ratio estimates with 95% confidence intervals. (Abbreviations: AoU: All of Us, CVD: Cardiovascular disease, AD: Alzheimer's disease, OR: Odds ratio)

CVD Subtype	OR	Lower Bound	Upper Bound
Hypotension	1.63	1.35	1.96
Cerebral.Infarction	1.59	1.27	1.98
Hypertension	1.42	1.17	1.72
Chronic.Ischemic.Heart.Disease	1.36	1.14	1.63
Heart.Failure	1.09	0.89	1.34
Cardiac.Arrhythmia	1.03	0.86	1.25
Angina.Pectoris	0.95	0.73	1.22

Chronic.Rheumatic.Heart.Disease	0.85	0.66	1.11
Acute.Myocardial.Infarction	0.75	0.55	1.01

Table S13 - Proximal SNPs related to the heart and brain. Traits are reported associated with SNP in the catalog, as well as traits that had significant UKB GWAS results, the number of pairings with each catalog SNP, and the gene(s) associated with each SNP. Only SNPs with greater than 2 pairings are reported. (Abbreviations: SNP: Single nucleotide polymorphism, UKB: UK Biobank, GWAS: Genome-wide association study, LV: Left ventricle, AHA: American Heart Association, MTAG: Multi-trait analysis of genome-wide association studies)

Catalog SNP	Catalog Traits	UKB Traits	Number of Pairings	Associated Genes
rs143364530	Alzheimer's disease or educational attainment (pleiotropy)	LV mean myocardial wall thickness AHA 11, LV mean myocardial wall thickness AHA 12, LV mean myocardial wall thickness global	42	*KANSL1*
rs12292911	Alzheimer's disease or family history of Alzheimer's disease	LV mean myocardial wall thickness AHA 5, LV mean myocardial wall thickness AHA 8, LV mean myocardial wall thickness global	23	*PSMC3, RAPSN*
rs4434960	Alzheimer's disease	LV mean myocardial wall thickness AHA 5, LV mean myocardial wall thickness AHA 8, LV mean myocardial wall thickness global	23	*PSMC3, RAPSN*
rs10437655	Alzheimer's disease (MTAG)	LV mean myocardial wall thickness AHA 5, LV mean myocardial wall thickness AHA 7, LV mean myocardial wall thickness AHA 8, LV mean myocardial wall thickness global	15	*SPI1*
rs73069394	Alzheimer's disease (MTAG)	Ascending aorta maximum area, Ascending aorta minimum area	10	*ULK4*
rs1065712	Alzheimer's disease	LV radial strain AHA 15	8	*CTSB*

rs3740688	Late-onset Alzheimer's disease	LV mean myocardial wall thickness AHA 5, LV mean myocardial wall thickness AHA 7, LV mean myocardial wall thickness AHA 8, LV mean myocardial wall thickness global	8	*SPI1*
rs4420638	total cholesterol measurement, hematocrit, stroke, ventricular rate measurement, body mass index, atrial fibrillation, high density lipoprotein cholesterol measurement, coronary artery disease, diastolic blood pressure, triglyceride measurement, systolic blood pressure, heart failure, diabetes mellitus, glucose measurement, mortality, cancer, total cholesterol measurement, diastolic blood pressure, triglyceride measurement, systolic blood pressure, hematocrit, ventricular rate measurement, glucose measurement, body mass	Alzheimer's disease, angina pectoris	7	*APOC1, APOC1P1*

	index, high density lipoprotein cholesterol measurement, Alzheimer disease, amyloid-beta measurement, Alzheimer's disease biomarker measurement, Alzheimer's disease biomarker measurement, t-tau measurement			
rs2696697	Alzheimer's disease polygenic risk score (upper quantile vs lower quantile)	LV mean myocardial wall thickness AHA 11, LV mean myocardial wall thickness AHA 12, LV mean myocardial wall thickness global	6	*KANSL1*
rs769449	coronary artery disease, diastolic blood pressure, Alzheimer disease, amyloid-beta measurement, t-tau measurement	Alzheimer's disease, angina pectoris	6	*APOE*
rs1065853	occlusion precerebral artery, systolic blood pressure, Alzheimer disease, polygenic risk score	Alzheimer's disease, angina pectoris	5	*APOE, APOC1*
rs429358	brain infarction, neuritic plaque measurement, Lewy body	Alzheimer's disease, angina pectoris	5	*APOE*

	dementia, cerebral amyloid angiopathy, neurofibrillary tangles measurement, systolic blood pressure, Alzheimer disease, Lewy body dementia, atrophic macular degeneration, age-related macular degeneration, wet macular degeneration, t-tau measurement			
rs157582	level of protein Wnt-10b in blood serum, Alzheimer disease, amyloid-beta measurement	Alzheimer's disease, angina pectoris	4	*TOMM40*
rs2075650	body fat percentage, coronary artery disease, posterior cortical atrophy, Alzheimer disease, t-tau measurement	Alzheimer's disease, angina pectoris	4	*TOMM40*
rs56131196	coronary artery disease, Alzheimer disease, Alzheimer disease, amyloid-beta measurement	Alzheimer's disease, angina pectoris	4	*APOC1, APOC1P1*
rs113568679	Alzheimer's disease or	LV mean myocardial wall thickness AHA 11,	3	*MAPT*

	educational attainment (pleiotropy)	LV mean myocardial wall thickness AHA 12, LV mean myocardial wall thickness global		
rs141622900	coronary artery disease, Alzheimer disease, high density lipoprotein cholesterol measurement	Alzheimer's disease, angina pectoris	3	*APOC1, APOC1P1*
rs199498	Alzheimer's disease	LV mean myocardial wall thickness AHA 11, LV mean myocardial wall thickness AHA 12, LV mean myocardial wall thickness global	3	*WNT3*
rs199503	Alzheimer's disease or educational attainment (pleiotropy)	LV mean myocardial wall thickness AHA 11, LV mean myocardial wall thickness AHA 12, LV mean myocardial wall thickness global	3	*WNT3*
rs199515	Alzheimer's disease	LV mean myocardial wall thickness AHA 11, LV mean myocardial wall thickness AHA 12, LV mean myocardial wall thickness global	3	*WNT3*
rs390082	level of protein S100-A13 in blood serum, Alzheimer disease	Alzheimer's disease, angina pectoris	3	*APOE, APOC1*
rs41290120	myocardial infarction, Alzheimer disease, high density lipoprotein cholesterol measurement	Alzheimer's disease, angina pectoris	3	*NECTIN2*
rs483082	Alzheimer disease,	angina pectoris	3	*APOE, APOC1*

	amyloid-beta measurement			
rs5117	lobar intracerebral hemorrhage, cerebral amyloid angiopathy	Alzheimer's disease, angina pectoris	3	*APOC1*
rs5167	level of hepatoma-derived growth factor-related protein 3 in blood serum, Alzheimer disease	Alzheimer's disease, angina pectoris	3	*APOC4-APOC2, APOC4*
rs59007384	Alzheimer disease, amyloid-beta measurement	angina pectoris	3	*TOMM40*
rs6859	cardiovascular disease, Alzheimer disease	Alzheimer's disease, angina pectoris	3	*NECTIN2*
rs7412	response to darapladib, lipoprotein-associated phospholipase A(2) change measurement, systolic blood pressure	Alzheimer's disease	3	*APOE*
rs814573	myocardial infarction, Alzheimer disease, polygenic risk score	Alzheimer's disease, angina pectoris	3	*APOC1, APOC1P1*

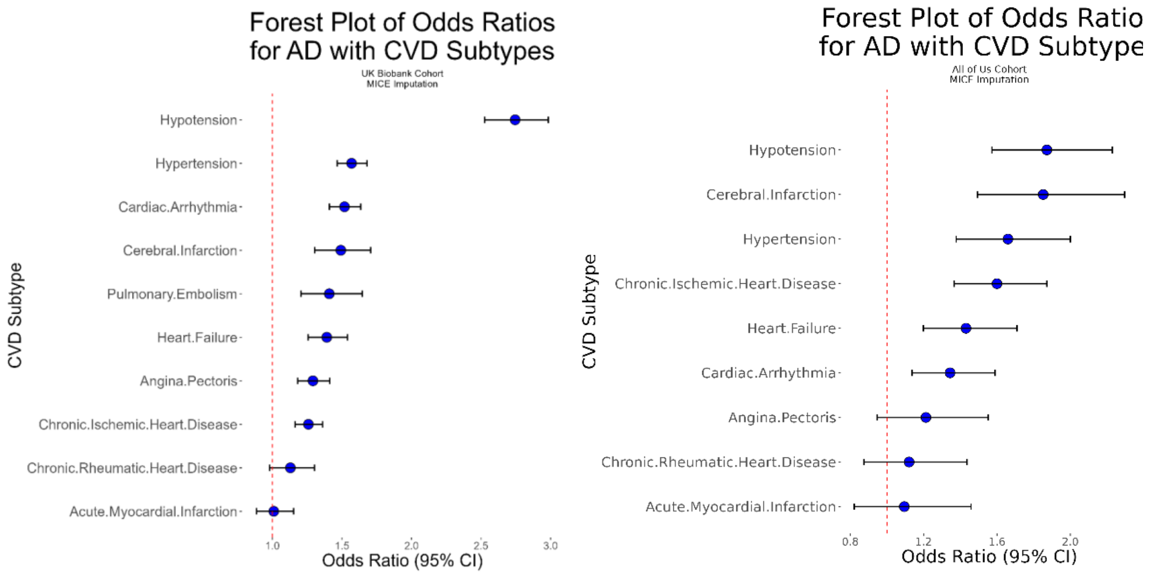

Figure S1 – CVD and AD odds ratios with MICE imputation. In both UKB and AoU, odds ratio estimates with MICE imputation were nearly identical to those obtained with imputed means, indicating that using the simpler method produces statistically valid results. (Abbreviations: AD: Alzheimer's disease, CVD: Cardiovascular disease, MICE: Multiple imputation by chained equations, CI: Confidence interval)

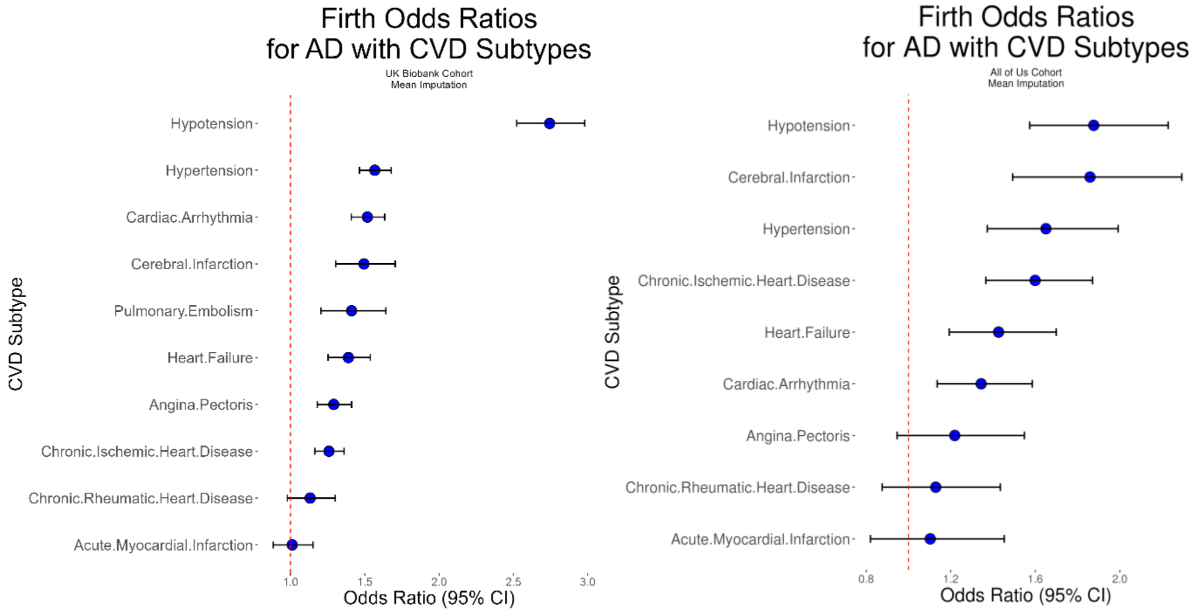

Figure S2 – CVD and AD odds ratios with Firth regression. In both UKB and AoU, odds ratios obtained with Firth regression were nearly identical to those obtained with simple logistic regression, indicating that odds ratio estimates are stable despite case-control

imbalances in some strata. (Abbreviations: AD: Alzheimer's disease, CVD: Cardiovascular disease, CI: Confidence interval, UKB: UK Biobank, AoU: All of Us)
